# A Comparison of English and Mandarin-Speaking Preschool Children’s Imitation of Motion Events

**DOI:** 10.3389/fpsyg.2017.01081

**Published:** 2017-06-28

**Authors:** Zhidan Wang, Haijing Wang

**Affiliations:** School of Educational Science, Jiangsu Normal UniversityXuzhou, China

**Keywords:** English, Mandarin, imitation, children, path, manner

## Abstract

Typically in English, a “satellite-framed” language, manner is expressed in the verb and path is expressed in supporting words. Past studies using looking time techniques suggest that English-speaking 3-year-olds show language-specific action processing, but 2.5-year-olds preferentially attend to path regardless of native language. In Study 1, we test whether language-specific action component preferences will be reflected in children’s imitation, as a more explicit measure. Children who spoke English saw an adult move an object along a series of platforms using one of two paths and manners. Then, the children were given the opportunity to move the object on a different test platform, which was designed to force them to choose to reproduce either the demonstrated path or the manner. The results showed that 3-year-olds, but not 2.5-year-olds, were more likely to imitate the manner versus the path. In Study 2, we extend the investigation to a less commonly studied language within this domain, Mandarin. Typically in Mandarin, an “equipollently framed” language, both manner and path are expressed within equally significant verbs. The results indicated that 3-year-olds did not show a consistent preference to imitate either the path or the manner. In contrast, 2.5-year-olds were more likely to imitate the path than the manner. This research highlights the potential for the imitation choice paradigm, as an explicit measure, to understand how language affects cognition, and suggests a new language-specific pattern in action interpretation.

## Introduction

Cognition and behavior are broadly influenced by culture. Language, specifically, is thought to establish “habitual patterns of thought” (Whorf, 1956). For example, the information that a language encodes in descriptions of motion events can lead to selective attention to path and manner elements of an event (e.g., Boroditsky, 2001). Adults prioritize these components reflects their linguistic experience (e.g., Naigles and Terrazas, 1998; Slobin, 2006). Recent investigations have begun to address this question from developmental perspective, primarily with looking time procedures. It has been suggested that regardless of native language, English-, Spanish-, and Japanese-speaking 2-year-olds preferentially attend to path, but 3-year-olds show language-specific action processing (e.g., Maguire et al., 2010). In Study 1, we apply a new methodology, the imitation choice paradigm, as an explicit measure to test the developmental change of English-speaking children in imitation of path and manner components during the preschool years. In Study 2, we extend the investigation of linguistic experience on action interpretation to a less commonly studied language within this domain, Mandarin.

**FIGURE 1:**
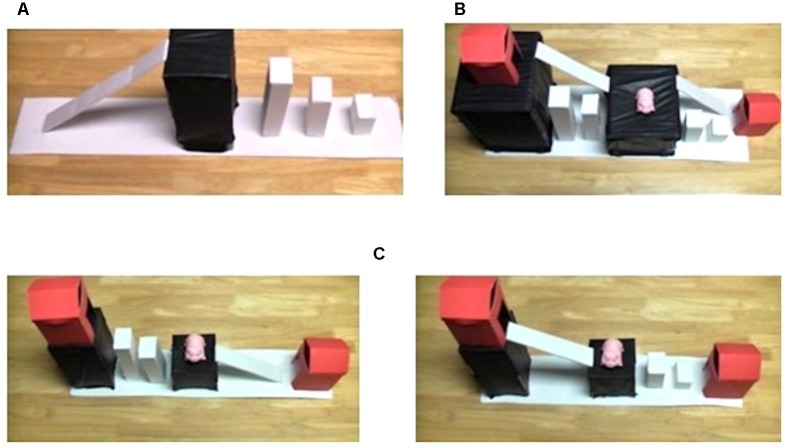
**(A)** Training phase apparatus. **(B)** Demonstration phase apparatus. Children saw the toy moved with one of two manners (hopping on pillars or sliding on ramps) and one of two paths (up or down) to one of the two houses. **(C)** Test phase apparatus. The test platforms contrasted path and manner relative to the adult’s demonstration.

A motion event typically includes four components; the primary object or actor (e.g., *a pig*), the path of motion (e.g., *up* or *down*), the manner (e.g., *hop* or *slide*), and the goal or end point of the event (e.g., *a house*). Within these, *path* and *manner* are so linguistically fundamental that languages are categorized typologically based on how they are encoded (Talmy, 1985, 2000). In ‘satellite-framed languages’ (S-languages), such as English, German, and Russian, manner is typically encoded within the verb itself, while the path of motion is instead encoded in other ‘satellite’ words supporting the verb, such as particles or prepositions (e.g., English The pig **hops** *up* to a house). In contrast, ‘verb-framed languages’ (V-languages), such as Spanish, Turkish, and Japanese, code the same path of motion within the verb by using an optional manner in the adverb (e.g., Spanish El cerdo ***salta*** a una casa/The pig **hops** *up* to a house) (**Figure 1**).

Adults consider the patterns found in their language when interpreting novel verbs (e.g., Naigles and Terrazas, 1998; Özçaliskan et al., 2003; Slobin, 2006). When presented with a motion event that involves both path and manner, English speakers interpret a language-specific novel verb as the manner used, whereas Spanish speakers encode it as the path taken (Naigles and Terrazas, 1998). Children begin to use and produce language-specific verb patterns at age 3. English-speaking 3-year-olds have been shown to use manner verbs significantly more than path verbs in their narratives, while Turkish-speaking children have been shown to use significantly more path verbs (Özçaliskan and Slobin, 1999). However, it seems that children first tend to construe a novel verb as referring to the path of the action regardless of their native language. Both Spanish- and English-speaking 2-year-olds produced more path expressions than manner expressions when they were beginning to talk (Naigles et al., 1992). In a nutshell, age 3 seems to be an important switch point in the use and production of language-specific verb patterns (Özçaliskan and Slobin, 1999; Özçaliskan, 2009; Chen and Guo, 2010).

Similar trends are seen in comprehension data by using looking time paradigm; English- and Spanish-speaking 2-year-olds demonstrate the same path preference when encoding motion verbs. However, the language-specific patterns emerge at age 3 years (Hohenstein et al., 2004; Mandler, 2006; Maguire and Dove, 2008). For example, Maguire et al. (2010) showed children a video clip including an animated object that moved using a distinctive path and manner (e.g., a starfish doing jumping jacks over a ball). A novel verb (e.g., *blicking*) was used to label the object’s activity. During the initial test, participants were asked to choose one of two new scenes as showing the referent of the same verb. In one scene, the object traveled along the old path, using a new manner. In the other scene, the object used the old manner, but moved along a new path. At age 3, language-specific differences were observed; English- and Spanish-speaking 3-year-olds chose the event showing the old manner paired with a new path, while Japanese-speaking children showed no preference in their construal of the novel verb. In contrast, 2- and 2.5-year-olds showed no difference as a function of their native language. Across English, Japanese, and Spanish, children interpreted the verb as referring to the path.

Empirical research has shown that subjects’ perceptions and conceptions of experimental stimuli are influenced by distinctions prominently marked in their native language (Boroditsky, 2001; Sera et al., 2002; Boroditsky et al., 2003). For example, Spanish adults are more likely to judge that two events that share the same path should be categorized together, while English speakers group events that share the same manner (Gennari et al., 2002). Researchers have begun to investigate the relationship between language and conceptual development from developmental perspective. Within the domain of action, some have argued that all children begin with similar conceptualizations. In particular, theorists have proposed that path is a core and obligatory component of a motion event (Talmy, 1985, 2000; Slobin, 2004). It is possible that all children, regardless of their native language, will be likely to encode and consider the path of a motion event (Mandler, 2006; Golinkoff and Hirsh-Pasek, 2008). As children come to recognize and practice the distinctions made in their own language, however, their interpretation of different motion events may change (Zheng and Goldin-Meadow, 2002).

To date, the majority of evidence in this realm comes from studies that measure infants’ looking time and use explicit language. The researchers employed the looking time paradigm by habituating infants to an event and then presenting them with a subsequent event that differed from the former one in a specific way in the test trial. They inferred that on average, infants look longer at the novel aspect of the second event. A benefit of the looking time paradigm is its high level of precision. However, the representations of behavior do not have to be especially robust within this paradigm because it tests behavior passively.

In the current research, we adapt the imitation choice paradigm designed by Wagner et al. (2008) to investigate how children represent the path and manner elements of a motion event. The imitation choice paradigm is a more demanding measurement of children’s processing priorities because it prompts children to represent what they observe, interpret it, and then reproduce it. Children not only have to use their memory to remember what they observe; they also have to put what they remember into action. Therefore, the imitation choice paradigm reflects deeper processing of an action than a looking time measurement does (Wagner et al., 2008).

An important way that young children learn about the world around them is from the acts of others. From very young ages, children are adept at imitating new acts that they see others perform. Those acts include opening containers, activating lights or sounds, and using simple tools (e. g., Meltzoff, 1988, 2007; Want and Harris, 2001; Carpenter et al., 2002; Nielsen, 2006). Although the basic elements of how to achieve a goal, like path and manner, are universal around the world, there may be differences in encoding these elements based on how this information is expressed in one’s own language. The proposed two studies are to examine whether children would vary their imitation of another’s path and manner depending on how this information is expressed within their native language of English or Mandarin.

In Study 1, we use children’s imitation as a test of their prioritization of different components of a motion event, with no explicit labeling used during the procedure. Specifically, an adult demonstrates an event that includes moving an object along one of two paths (*up* or *down*) using one of two manners (*hop* or *slide*) to reach a goal. During the test period, children were given the opportunity to move the object on a different test platform. The test platform was designed to force children to choose to replicate either the demonstrated path or the manner. The children’s imitation choices serve as a measure of the importance of each of the motion elements in their representation of the action. In line with past findings, we hypothesize that for English-speaking children, language-specific imitation patterns will emerge at age 3. Specifically, we predict that English-speaking 3-year-olds will be more likely to pick out and imitate the demonstrated manner versus path.

In Study 2, we test Mandarin-speaking children to explore this developmental question from a cross-linguistic perspective. In the typology of Mandarin Chinese, two verbs, one describing path and one describing manner, are necessary to describe a motion event (e.g., 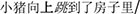
The pig **hops** *up* to a house). Recently, Slobin (2004) described Mandarin as an “equipollently framed” language, in which both manner and path are expressed by two elements of equal importance. To date, how this typological system affects thought has received relatively little attention in the literature. If the roles of path and manner are equal in everyday expression, this may influence the way speakers encode a motion event explicitly. Specifically, we hypothesize that Mandarin-speaking 3-year-olds will not show a consistent preference to imitate the path or the manner of the demonstration. In a cross-study comparison, we predicted that Mandarin-speaking children would be less likely to show a strong preference for imitating either manner or path across the trials than English-speaking children.

## Study 1

The objective of Study 1 was to examine which element was the children’s priority when they were allowed to imitate the demonstrated manner versus the path in the English-speaking 2.5- and 3-year-olds. We predicted that English-speaking 3-year-olds would be more likely to imitate the demonstrated manner than the path, reflecting their language-specific verb processing patterns.

### Materials and Methods

#### Participants

Sixteen 2.5-year-olds (ranging from 27 to 34 months, *M* = 30.1 months, *SD* = 1.82 months; 8 males) and nineteen 3-year-olds (ranging from 36 to 40 months, *M* = 36.4 months, *SD* = 1.12 months; 10 males) participated in the experiment^1^. The children were recruited by telephone calls from the “Infant and Child Subject Database” which has been approved by Georgia State’s institutional review board (IRB). This database is designed to include a diverse sample of families from the Atlanta metro area who have already expressed interest in participating in research studies at Georgia State University. According to parental report, the final sample was 70% white, 20% Black/African American, 5% mixed race, and 5% not reporting. All children in the final sample came from monolingual English families, with English being the only language spoken to children since birth.

#### Materials

The materials included a small pink pig toy (7 cm × 3 cm × 5cm) that the experimenter moved across ramps and pillars to platforms at different levels. The *training phase apparatus* (75 cm × 13 cm × 23cm) allowed the experimenter to demonstrate both of the manners and paths to be used in the study. This apparatus included a ramp (on which the pig was slid) and a set of pillars (on which the pig was hopped), both leading up to a platform (**Figure 1A**).

The *demonstration phase apparatus* (75 cm × 24 cm × 30cm; **Figure 1B**) allowed the experimenter to present any combination of path and manner. This apparatus had three platforms (the tabletop, a middle level, and a top level.) The middle level platform was connected to the tabletop and the top-level platform by *both* a ramp and a set of pillars (**Figure 1B**). The tabletop and top-level platform each had a red house (9 cm × 9 cm × 13cm) that the pig could fit into.

For the testing phase, two additional apparatuses (64 cm × 13 cm × 23cm) were developed. Each also consisted of three platforms (the tabletop, a middle level, and a top level), and a house was placed on the tabletop and the top level (**Figure 1C**). In contrast to the demonstration phase apparatus, the tabletop and the top-level platforms were connected to the middle platform by *either* a ramp or a set of pillars, but not both. These platform sets were designed to contrast path and manner elements presented on the demonstration phase apparatus.

#### Procedure

Children were tested individually in the Laboratory at the Georgia State University, and their behaviors were videotaped for subsequent scoring. Informed consent from children’s parents or legal guardian(s) was received prior to testing. Each testing session included three phases. The total testing session took approximately 5 min.

##### Training phase

The goal of this phase was to familiarize children with the different actions used in the experiment. Children saw the experimenter move the toy on the training phase apparatus up from the tabletop, to the middle platform, and then down to tabletop on the other side of the platform. Then, the experimenter turned the toy around and moved the along the apparatus back to the original location. In this way, the experimenter moved the toy along each path (up, down) using each of the manners (sliding on the ramp, hopping on the pillars). Two orders were developed for the presentation of these acts, and which was used was counterbalanced between children. After this phase, children were introduced to the demonstration materials.

##### Experimental trials

There were four experimental trials, each consisting of a demonstration and a test phase. Each trial used a different manner/path combination during the demonstration (i.e., *hop up*, *hop down*, *slide up*, or *slide down*). Trials were presented in one of four orders, and which order was chosen was counterbalanced between the children.

##### Demonstration phase

During this phase, the experimenter showed the children an action event that they would later be given the opportunity to imitate. To minimize the impact of the verbal input, minimal instructions were provided. The experimenter drew the children’s attention, “Now let’s play the game. It’s my turn first, and then it’s your turn.” If necessary, children were prompted with general statements such as, “Now it is your turn to play the game. Here you go.” No verbal descriptions of the manner or path were ever provided. Children saw the experimenter move the small toy from the middle platform to a red house using one manner and path (e.g., the pig was hopped down a set of pillars to a house).

##### Test phase

After each demonstration, the experimenter placed the toy in the middle platform of one of the testing apparatuses. Which testing platform set was chosen depended on the movement used during the demonstration phase. For example, if the experimenter hopped the pig down during demonstration, the testing platform that had pillars going up and a slide going down from the middle platform was used. Thus, the materials were designed to lead the children to match the demonstrated manner (by *hopping* up) or the demonstrated path (by *sliding down*), but not both. The children were encouraged to move the toy from the center platform (e.g., “Now it is your turn to play with these. Here you go”). The trial ended when the children moved the toy into one of the two houses. Then, the experimenter began the next trial with a new demonstration.

##### Dependent measures and scoring

Although we designed the test materials to lead children to choose either the path or the manner, children did not always use the appropriate movements for the platforms. For example, they sometimes hopped the pig on the slide. We therefore scored children’s first movement of the toy into the house as using one of four different imitative strategies (as in Wagner et al., 2008): (a) if the child reproduced the path at the expense of the manner, he/she was scored as 1 for his/her path imitation score but 0 for his/her manner imitation score; (b) if the child reproduced the manner at the expense of the path, he/she was scored as 1 for his/her manner imitation score but 0 for his/her path imitation score; (c) if the child reproduced both the path and the manner, he/she was scored as 1 for both his/her path and manner imitation score; (d) if the child reproduced neither the path nor the manner, he/she was scored as 0 for both his/her path and manner imitation score. The sum of the four trials resulted in a path imitation score and a manner imitation score, both of which ranged from 0 to 4 for each child. We calculated a *component preference score* by using manner imitation score minus path imitation score, with positive scores indicating more imitation of the manner elements relative to path and negative scores more imitation of the path elements relative to manner.

The primary scorer was a research assistant who remained uninformed of the participant’s group assignment and the study hypotheses. A second scorer, also unaware of group assignment, coded a randomly selected 25% of the participants (Cohen’s κ = 0.89).

### Results and Discussion

Preliminary analysis showed no significant effects of gender on the component preference score, *t*(33) = 0.41, *p* = 0.68, *d* = 0.14. Non-parametric Friedman test was conducted to examine the effect of the trial order on the component preference score. The results indicated that there was no effect of the trial order, χ^2^(3, *N* = 35) = 3.05, *p* = 0.38. We collapsed across these two factors in the subsequent analyses.

**FIGURE 2:**
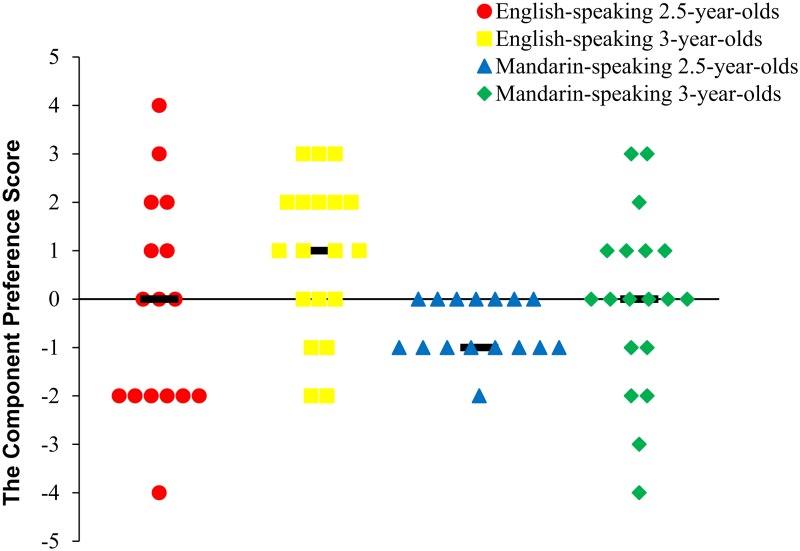
Univariate scatter-plots for the component preference score as a function of age and the children’s first language.

Our hypothesis was that the language-specific imitation patterns of English-speaking children will emerge around age 3 years, with increased imitation of manner. To evaluate this hypothesis, one-sample *t*-test indicated that the component preference score of the 3-year-olds (*M* = 0.90, *SD* = 1.60) was significantly above 0, *t*(18) = 2.45, *p* = 0.03, *d* = 1.15, indicating more imitation of the demonstrated manner versus the demonstrated path. In contrast, the component preference score of 2.5-year-olds (*M* = -0.19, *SD* = 2.23) was not significantly different from 0, *t*(15) = -0.34, *p* = 0.74, *d* = -0.18, indicating that they were equally likely to imitate the path and the manner. These results suggested that there was a developmental change in English-speaking children’s imitation of the manner versus the path between the ages of 2 and 3 years. As shown in **Figure 2**, we provide univariate scatter-plots instead of bar graphs, especially given the small sample sizes in the current study (Weissgerber et al., 2015).

## Study 2

The objective of Study 2 was twofold. First, we examined whether the developmental change observed in English-speaking children would also be shown in Mandarin-speaking children. Specifically, whether the language-specific imitation patterns of Mandarin-speakers would also emerge at age 3. Second, we compared Mandarin-speaking children in Study 2 to the English-speaking children in Study 1 to examine whether there was a difference in children’s imitation of motion events.

### Materials and Methods

#### Participants

Sixteen 2.5-year-olds (ranging from 26 to 32 months, *M* = 30.3 months, *SD* = 1.94 months; 8 males) and nineteen 3-year-olds (ranging from 36 to 42 months, *M* = 39.0 months, *SD* = 1.99 months; 8 males) participated in the experiment. The sample size was determined based on the number of United States American children that were tested. Children were recruited from a preschool which attached to an university in a mid-sized city in China. Permission to test the children was obtained from the teachers. The children were predominantly Han ethnicity and live in areas surrounding the university. All the children included in the final sample came from monolingual Mandarin Chinese families, with Mandarin being the only language spoken to them since birth. Georgia State University’s IRB provided oversight of the project.

#### Materials

The experimental stimuli used in Study 1 were used again in Study 2.

#### Procedure

The general procedure was identical to that in Study 1 except that children were tested individually in a quiet room at their school. Children’s responses were recorded online by two research assistants because we did not have permission to video tape the Chinese children.

##### Dependent measures and scoring

The dependent measures were identical to those in Study 1.

The children’s responses were recorded online by two research assistants. They initially were blind to the specific hypotheses of the study. The interrater reliability between the two research assistants also was assessed using the Cohen’s kappa (0.92).

### Results and Discussion

Preliminary analysis also showed no significant effects of gender on the component preference score, *t*(33) = -0.41, *p* = 0.69, *d* = -0.14. Non-parametric Friedman test suggested that there was no effect of the trial order on the component preference score, χ^2^(3, *N* = 35) = 5.28, *p* = 0.15. We collapsed across these factors in the subsequent analyses.

One-sample *t*-test indicated that the component preference score of the Mandarin-speaking 3-year-olds (*M* = -0.05, *SD* = 1.79) was not significantly above 0, *t*(18) = -0.13, *p* = 0.90, *d* = -0.06. It suggested that Mandarin-speaking 3-year-olds were equally likely to imitate the path and the manner. However, the component preference score of Mandarin-speaking 2.5-year-olds (*M* = -0.63, *SD* = 0.70) was significantly below 0, *t*(15) = -4.04, *p* = 0.001, *d* = -1.99, indicating more imitation of the demonstrated path versus the manner. These results suggested that there was a developmental change in Mandarin-speaking children’s imitation of the demonstrated elements between the ages of 2 and 3 years (**Figure 2**).

We conducted an univariate ANOVA with language (English vs. Mandarin) and age (2.5- vs. 3-year-olds) as between-subject factors on children’s *component preference score* collected from English-speaking children in Study 1 with that of Mandarin-speaking children in Study 2. This analysis showed a main effect of age, *F*(1,70) = 4.20, *p* = 0.04, $\eta_{p}^{2}$ = 0.06. This indicates the 3-year-olds (*M* = 0.42, *SD* = 1.77) were more likely to imitated the demonstrated manner versus the path. In contrast, the 2.5-year-olds (*M* = -0.41, *SD* = 1.62) were more likely to imitated the demonstrated path versus the manner. There was a marginally significant main effect of language, *F*(1,70) = 2.94, *p* = 0.09, $\eta_{p}^{2}$ = 0.04, suggesting the English-speaking children (*M* = 0.40, *SD* = 1.96) were more likely to imitated the demonstrated manner versus the path. In contrast, the Mandarin-speaking children (*M* = -0.31, *SD* = 1.43) were more likely to imitated the demonstrated path versus the manner. However, the results showed that there was no significant age × language interaction, *F*(2,69) = 0.40, *p* = 0.53, $\eta_{p}^{2}$ = 0.006.

### Exploring Language-Specific Effects Further

There were no significant differences in the Mandarin-speaking and English-speaking children’s component preference scores. However, there was a cultural difference in the number of children that showed a strong individual preference for imitating either the demonstrated manner or the path. Children were grouped according to their component preference score to determine whether individual children showed a strong preference for imitating either manner or path across the trials. Group one included children who showed a strong preference for imitating manner (2 ≤ component preference score ≤ 4) or path (-4 ≤ component preference score ≤-2). Group two included children who did not exclusively imitate path or manner on more than 50% of the trials (-1 ≤ component matching score ≤ 1). An non-parametric chi-square test was performed to compare the component preference score. The results showed that English-speaking children (21/35) were more likely than Mandarin-speaking children (8/35) to show a strong preference for imitating one element (either path or manner) of the demonstration, χ^2^(1, *N* = 70) = 9.95, *p* = 0.002, *V* = 0.37.

## General Discussion

In this research, English- and Mandarin-speaking 2.5- and 3-year-olds were shown an adult moving an object along a path in a specific manner. During the test phase, the children were given different test platforms that contrasted the path versus the manner relative to the adult’s demonstration. The children’s reproductions of the path and manner components of the movement were then evaluated. This research contributes three findings to the literature on how language may influence thought. First, we found that English-speaking 3-year-olds, but not 2.5-year-olds, were more likely to imitate the manner versus the path. Second, Mandarin-speaking 2.5-year-olds were more likely to imitate the path versus the manner, while 3-year-olds did not show a consistent preference for imitating either the path or the manner. Third, English-speaking children were more likely than Mandarin-speaking children to show a strong preference for imitating one element of the demonstration.

In Study 1, the observed developmental change in English-speaking children’s language-specific pattern in the interpretation of action is in line with past findings, which established a similar developmental change between 2 and 3 years of age by using the looking time paradigm (Golinkoff and Hirsh-Pasek, 2008; Maguire et al., 2010). For example, English-speaking 3-year-olds, but not 2-year-olds, show a language-specific action preference for manner (Maguire et al., 2010). These results are consistent with several other studies that use a narrative speaking approach (Naigles et al., 1992; Özçaliskan and Slobin, 1999). Notably, the current studies provide the first experimental evidence for this developmental change by using an explicit measure of children’s representation of action—specifically their imitation of another’s acts.

2.5-year-olds did not demonstrate the use of language-specific action processing patterns. This may be the case because children may not use their language’s typology to guide their verb construction by age 2 (Mandler, 2006; Golinkoff and Hirsh-Pasek, 2008). As they gain more experience (e.g., vocabulary) with their language, their preference emerges and they begin to demonstrate a language-specific approach (Zheng and Goldin-Meadow, 2002). Another possibility is that as they grow older, the systematic and repeated expression of certain aspects of spatial scenes and events in language direct children’s attention and thought to one specific language element more than others (Peterson et al., 1996; Li and Gleitman, 2002; Gentner and Goldin-Meadow, 2004).

In Study 2, there was also a developmental change observed in Mandarin-speaking children. Mandarin-speaking 2.5-year-olds were more likely to imitate the demonstrated path. It is possible that this is because path is the core and obligatory component of a motion event (Talmy, 1985, 2000; Slobin, 2004). Paths were also categorized earlier than manners (Pruden et al., 2005). In the domain of cognitive psychology, path is used as the basis element for event segmentation (Shipley and Maguire, 2008), and changes in path always indicate information about when events begin and end (Mandler, 2004).

Another contribution of the current research is the possibility of a new language-specific pattern in the interpretation of action. Mandarin-speaking 3-year-olds were equally likely to imitate path and manner. Recall that the cross-study comparison also showed that in contrast to the majority of English-speaking children who consistently imitated manner or path across the testing trials, the majority of individual Mandarin-speaking children showed no strong preference to imitate path or manner across trials. The Mandarin-speaking children’s pattern of imitation may reflect their language’s equal emphasis on path and manner, as in the description of Mandarin as an “equipollently framed” language (Slobin, 2004; Ji, 2009; Chen and Guo, 2010).

This research highlights the potential for the imitation choice paradigm to contribute to our understanding of how language affects cognition. From very young ages, children imitate other acts (Meltzoff, 1988, 1995; Carpenter et al., 2002; Nielsen, 2006), and imitation measures are increasingly being used through childhood and even into adulthood (McGuigan et al., 2011). Thus, the imitation choice paradigm is a non-verbal test of a participant’s action representation that can be used across a variety of ages.

This experiment also contributes to our understanding of children’s interpretation and reproduction of action. Children can imitate many aspects of a demonstration. 14-month-olds have been shown to imitate both goals and distinct manners used by adults (Meltzoff, 1988). For example, they will turn on a light panel (goal) by pressing with their forehead (manner) if they see an adult do this. It has been shown that children are flexible and selective imitators. They do not simply copy all of the behaviors of adults, they choose what to imitate according to several factors. For one, when there is a clear goal, children are less likely to imitate the manners used by an adult (Bekkering et al., 2000; Carpenter et al., 2005; Williamson and Markman, 2006). 12- and 18-month-olds were likely to imitate moving a toy mouse in a house (goal) but to overlook the hopping motion and the sound effects that the adult used (manner) (Carpenter et al., 2005). Another factor shown to influence children’s imitation is whether the manners appear to be rational. Gergely et al. (2002) made a little change to Meltzoff’s (1988) study-when the adult’s hands were occupied by a blanket, children tended to push the light-box with their hands instead of imitating the adult’s use of the forehead.

This research is designed to test whether different first languages correspond to differences in children’s imitation. The results lead to the conclusion that children may prioritize components of actions that lead to a goal and that this prioritization may vary depending on their linguistic experience. Specifically, English-speaking children were more likely to imitate the manner than were Mandarin-speaking children. In English, manner is encoded with the main verb itself. In contrast, both path and manner are encoded in the verb in Mandarin. Children’s imitation of the path and manner may reflect how this information is expressed in their native language. It not only deepens our understanding of children’s imitative behavior from a cross-linguistic perspective but also contributes to our understanding of children’s social learning processes more generally.

### Limitations and Future Directions

Future research should address and try to improve upon the limitations that are present in this research. It should be noted that the current research presents only a first step in investigating how linguistic experience may influence imitation. There is no denying that there are many differences between Chinese and American cultures that do not involve language. Although the observed age-related changes fit with current theories of the role of language in shaping cognition, this replication in a wider sample of cultures in the future is necessary to pinpoint language as the primary factor driving the observed results. We also recognize the need for caution in interpreting the results of this research given the small sample size and the differences between the two experimental settings.

One limitation in the methodology of the current research is that the spontaneous responses of children when they are allowed to interact with the stimuli are not directly assessed. A baseline control group in which children see no demonstration before attempting the tasks would be a helpful addition. This information would help us determine whether true imitation has taken place. In addition, only one imitation task was used in our study, and as a result, we cannot assess whether children would also show the developmental change observed in this study when given similar imitative tasks. One way to reduce this limitation is to use two or more tasks and test children once on each task. If the current results were replicated, we could reach a conclusion more confidently.

Another limitation of the current study is we did not test children from a V-language background. An important future step is to test the imitation of path and manner in children who speak a typical V-language in which path is typically encoded in the verb, while manner is often optional, such as Turkish or Spanish. If both 2- and 3-year-olds show more imitation of path versus manner, it would lend support to the proposal that language, and not a general development, is driving the observed change. In addition, we also did not test children’s preferences by using the looking time paradigm. In a future study, we will show children one video of a particular event, such as a pig hopping up to a house. Then, we will show them two testing videos on the screen side by side. In one video, the pig will hop down to a house, whereas in the other video, the pig will slide up into a house. It will be interesting to examine whether we will find a similar pattern to that found by the current study based on children’s looking preferences.

## Conclusion

This research addresses whether linguistic typology influences children’s imitation of motion events. These results add to the growing body of literature showing the developmental shift in children’s path and manner preference between 2.5 and 3 years of age by using a more explicit measure-imitation choice paradigm. The application of this paradigm to the examination of the linguistic typological questions posed by this study helps us to understand them through an innovative methodology. In addition, the inclusion of Mandarin, a less commonly studied language within this discipline, enabled us to gain a broader understanding of how linguistic typology may influence action interpretation across languages. In conclusion, these findings have deepened our understanding of how development and language influence children’s imitation of motion events.

## Author Contributions

ZW designed the experiments, wrote the paper, and analyzed the data. HW performed the experiments and wrote the paper.

## Conflict of Interest Statement

The authors declare that the research was conducted in the absence of any commercial or financial relationships that could be construed as a potential conflict of interest.

## Footnotes

1 One 3-year-old child’s data was excluded due to video error. Four 2-year-olds were tested but not included in the final sample because they did not complete all the four testing trials.
